# Minimally Invasive Spinal Surgery with Intraoperative Image-Guided Navigation

**DOI:** 10.1155/2016/5716235

**Published:** 2016-04-24

**Authors:** Terrence T. Kim, J. Patrick Johnson, Robert Pashman, Doniel Drazin

**Affiliations:** ^1^Department of Orthopedics, Cedars-Sinai Medical Center, Los Angeles, CA 90048, USA; ^2^Department of Neurosurgery, Cedars-Sinai Medical Center, Los Angeles, CA 90048, USA; ^3^Department of Neurosurgery, University of California Davis Medical Center, Sacramento, CA, USA

## Abstract

We present our perioperative minimally invasive spine surgery technique using intraoperative computed tomography image-guided navigation for the treatment of various lumbar spine pathologies. We present an illustrative case of a patient undergoing minimally invasive percutaneous posterior spinal fusion assisted by the O-arm system with navigation. We discuss the literature and the advantages of the technique over fluoroscopic imaging methods: lower occupational radiation exposure for operative room personnel, reduced need for postoperative imaging, and decreased revision rates. Most importantly, we demonstrate that use of intraoperative cone beam CT image-guided navigation has been reported to increase accuracy.

## 1. Introduction

Neurological sequelae may result from pedicle screw misplacement during spinal instrumentation and fusion, and inaccurate placement can be fairly frequent with conventional fluoroscopy [1–3]. Because of limited dissection and exposure during spine operations and especially with minimally invasive spine surgery (MISS) techniques, spine surgeons have heavily relied on intraoperative fluoroscopy for procedures such as pedicle screw insertion [4]. However, this has raised concerns over the level of radiation exposure for the all persons in the operating room.

The drawbacks associated with conventional intraoperative imaging methods have increased the interest in improving navigation methods in spine surgery, evolving tremendously over the last few years [5–12]. Use of 2-dimensional (2D) fluoroscopic navigation moderately decreased the number of improperly placed screws and introduction of preoperative CT scan imaging navigation techniques further reduced the percentage [13–15]. Such computer-based navigation technology has facilitated complex procedures in MISS, where visualization is limited. The use of 2D and 3D navigation systems during the last decade has made spine operations safer and less invasive [6, 16, 17]. Many authors have highlighted the use of image navigation for spinal operations as a way to decrease radiation exposure and operative time [18–20].

Most recently, introduction of the O-arm (Medtronic, Inc.) has allowed spine surgeons to perform minimally invasive procedures accurately, safely, and more efficiently [6]. The O-arm is a 3-dimensional (3D) imaging system that provides full 360° rotational capability that can interface with an external navigation system [21, 22]. While providing excellent imaging and navigation to guide an operation, the O-arm also permits the surgeon to obtain immediate CT images at the completion of surgery [5]. This would allow for immediate intervention if necessary before closure. Others have highlighted the O-arm's capability to obtain CT images with multicut reconstructions along with navigation to make it ideally suitable for MISS [21, 23].

In a recent international, multicenter, prospective study over a 16-month period, 353 patients underwent 1922 pedicle screw placements using the O-arm [5]. It was determined that 2.5% of the screws were misplaced and mean patient radiation dose was equivalent to half the dose of a 64-multislice CT scan [5]. Though the study contained a small number of MIS cases, the authors suggested that future studies should evaluate the O-arm in a large series of MIS procedures.

In this paper, we describe our perioperative MIS technique for placing percutaneous spinal instrumentation utilizing the O-arm with navigation.

## 2. Surgical Technique and Pearls

### 2.1. Patient Positioning

All O-arm MISS patients were placed prone on a Jackson radiolucent spinal operating table, and all pressure points were padded appropriately. The dorsal lumbar spine was sterilely prepped and care was taken to drape the entire lumbar spine as wide as possible. The authors recommend having a wide draping area as it allows for skin surface anatomy identification (posterior superior iliac spine, iliac crest, and midline spinous processes) and gives orientation during the entire procedure.

### 2.2. Navigated Reference Frame

**Figure 1:**
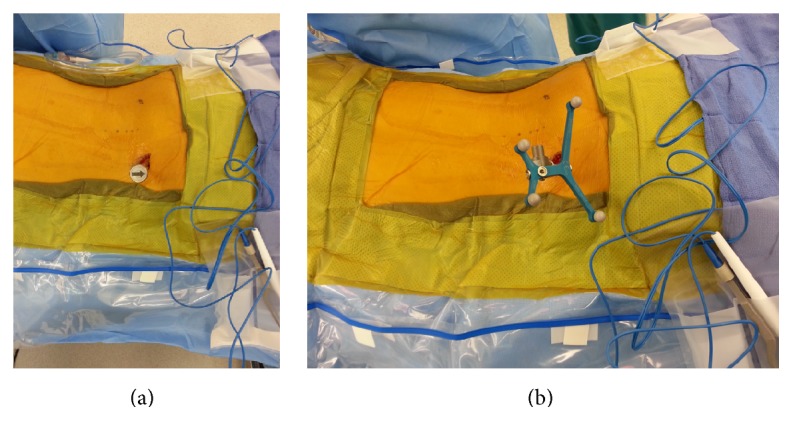
Intraoperative images of the percutaneously placed reference pin and attached navigation frame in the left ilium inferior to the level of the posterior superior iliac spine (PSIS).

Selection of ideal reference frame and placement are dependent on the goals of the surgeon and anatomy of the patient. A percutaneous reference pin was routinely placed in the left ilium inferior to the level of the posterior superior iliac spine for short-segment percutaneous pedicle screw instrumentation (Figures 1(a) and 1(b)). For MISS in the lower lumbar spine (L3-S1), we found that the posterior superior iliac spine (PSIS) reference frame oftentimes interfered with the trajectory and instruments for insertion of the ipsilateral pedicle screws. As a result, in these instances, we would select a midline percutaneous incision directly over a proximal spinous process and use the navigated spinous process clamp reference frame directed away from the surgical area. Placement of the StealthStation workstation (Medtronic, Inc.) and StealthStation (LED detector camera) was placed at the foot of the bed for PSIS frames and head of the bed for the midline spinous process reference frame. Careful attention to “line-of-sight” issues for the navigated reference frame and StealthStation placement in the room are two examples of instrument issues that a surgeon must consider in order to maximize the workflow for a navigated MISS.

### 2.3. CT Image Acquisition

Three-dimensional CT images were obtained using a cone beam mobile CT scanner (O-arm, Medtronic, Inc.) and were transferred to the computer-assisted StealthStation surgical navigation workstation. All navigated probes and instrumentation were calibrated. Although it is possible to keep the O-arm in the sterile surgical field during the entire procedure, it was our preference to remove the O-arm and station it in the operating room.

### 2.4. Incision

**Figure 2:**
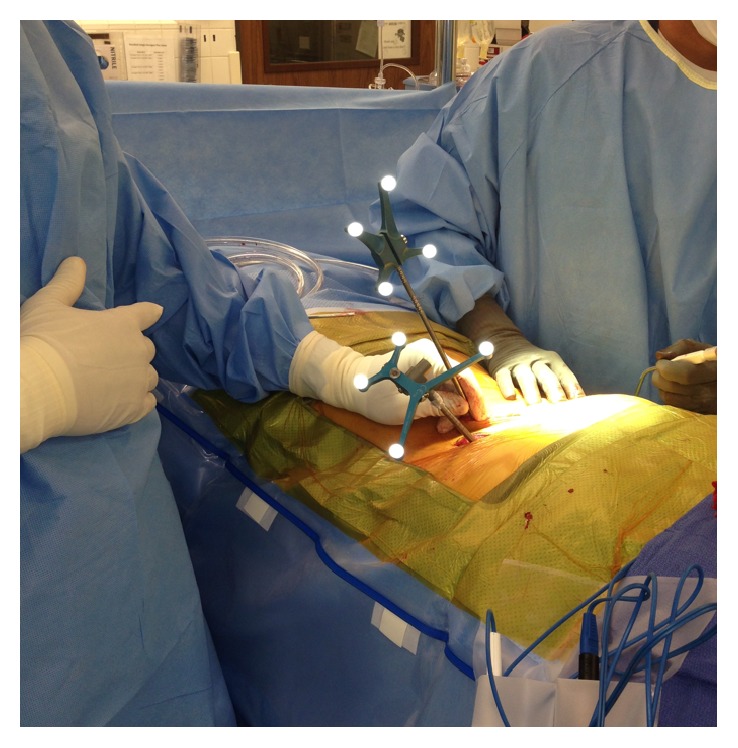
Intraoperative images demonstrating the use of the navigated probe to plan out the paramedian laterally based skin incisions.

Paramedian laterally based skin incisions were planned out using a navigated probe (Figure 2). Planning pedicle screw trajectories using the navigated probe with “forward-projection” on the StealthStation allowed for accurate placement and incisional length. For patients where a midline incision was necessary (in the case of decompression), subfascial exposure or separate percutaneous paramedian skin incisions were selected for pedicle screw insertion.

### 2.5. Placement of Spinous Process Pin (Optional)

**Figure 3:**
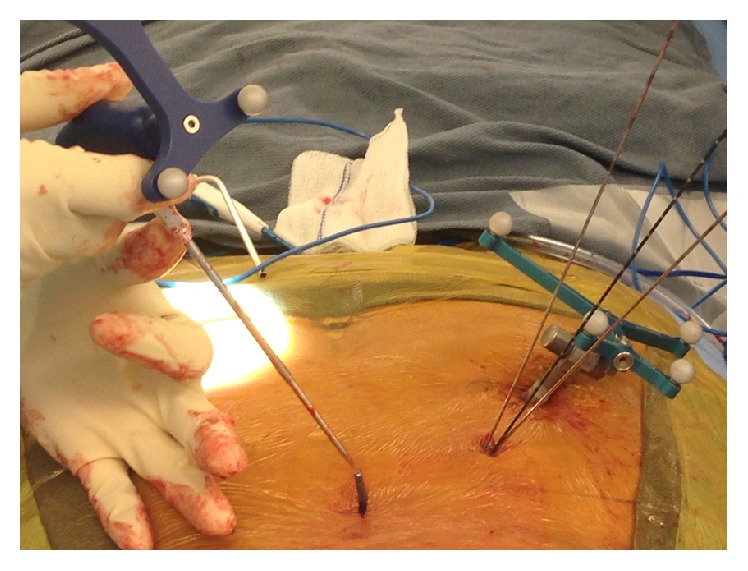
Intraoperative image demonstrating the Caspar pin that was inserted, prior to image acquisition, into the bony spinous process to enable the surgeon to use this pin as a reliable checkpoint for accuracy for all navigated instruments.

In our earlier experience with PSIS percutaneous reference frames, a Caspar pin was directly inserted through a percutaneous midline incision into one of the spinous processes within the acquired CT image. In MISS navigation surgery, there is oftentimes no clear anatomic point that can be referenced to determine navigation accuracy. Especially in morbidly obese patients, bony surface anatomy can be hard to palpate and assess. By obtaining a CT image with the Caspar pin inserted into the bony spinous process, the surgeon can reliably use this point as a check for accuracy for all navigated instruments (Figure 3).

### 2.6. Navigated Insertion of Instrumentation

**Figure 4:**
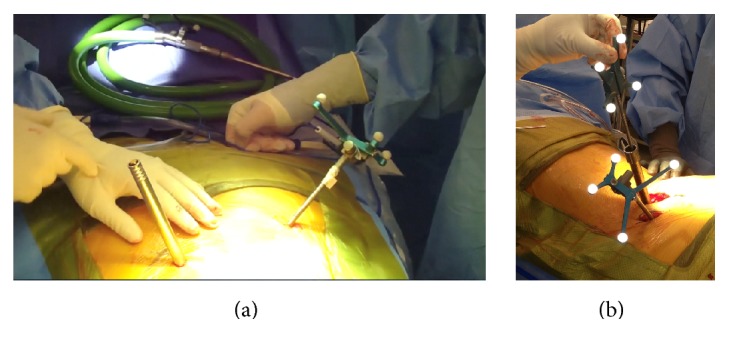
Intraoperative images demonstrating navigated dilators inserted through the paraspinal muscle and docked onto the ideal pedicle screw starting point as determined by navigation.

**Figure 5:**
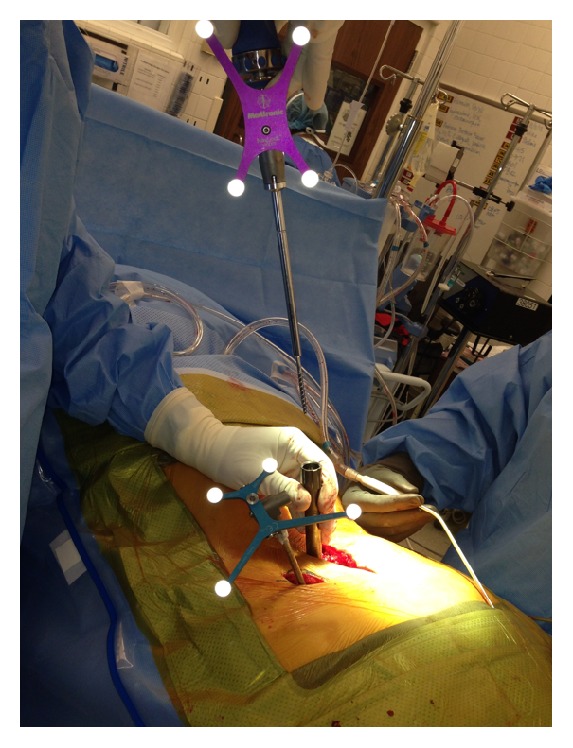
A navigated cannulated awl is passed through the soft tissue dilator and enters the center of the pedicle as determined by navigation.

**Figure 6:**
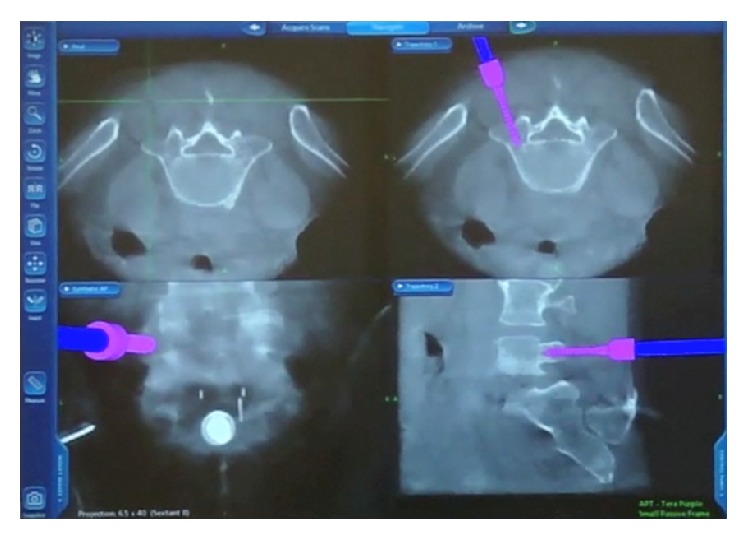
StealthStation computer screen projection of a pedicle screw being inserted into the pedicle with a navigated driver.

A navigated dilator was inserted through the Wiltse paraspinal muscle interval and docked onto the ideal pedicle screw starting point—lateral to the facet joint (Figures 4(a) and 4(b)). Serial dilators were inserted and a navigated cannulated awl (Jamshidi) was used to enter the center of the bony pedicle canal as determined by the navigated axial, coronal, and sagittal planes (Figure 5). Precise placement of the initial Jamshidi is critical to achieving high accuracy with navigated MISS pedicle screws. Anatomic variances of dorsal vertebral bone surfaces and pedicle starting points can be irregular and difficult to dock. We found that it was critical to insert the navigated Jamshidi awl in a very lateral-to-medial starting trajectory in order to minimize “slipping” off the starting point. In addition, we emphasize inserting the Jamshidi with minimal pulling/pushing of surrounding soft tissue. Our technique emphasizes the concept of inserting the Jamshidi through the paraspinal muscles like “throwing a dart.” Minimizing soft tissue retraction and manipulation minimizes variance in navigation trajectory and ultimately translates into more accurate pedicle screws. Guide wires were inserted through the cannulated Jamshidi to maintain pedicle trajectory and position. Once the pedicle was cannulated, a navigated tap was used to tap the bony pedicle tract and the desired size and length screw was determined via the StealthStation measurements. The largest sized pedicle screw with 1-2 mm of circumferential bony containment was finally inserted into the pedicle with a navigated screwdriver and confirmed on the StealthStation computer screw projection (Figure 6). It is noted that significant downward forces during tap cannulation and screw insertion can be applied to the vertebral body, thereby moving the vertebral body from its original imaged position. This can lead to significant inaccuracy and misplaced instrumentation. Current navigation technology is unable to account for shifts in vertebral body position and dynamic change in the spinal bony anatomy. As a result, we recommend extreme care in minimizing extreme forces that would displace or alter the alignment of the operated spine.

**Figure 7:**
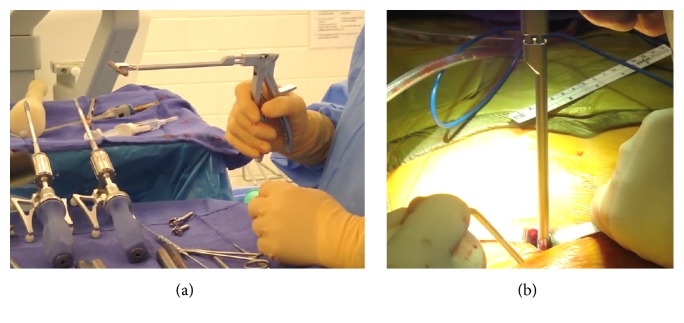
Intraoperative images of a short-segment lumbar fixation case demonstrating insertion of the rod under direct visualization using a rod holder.

**Figure 8:**
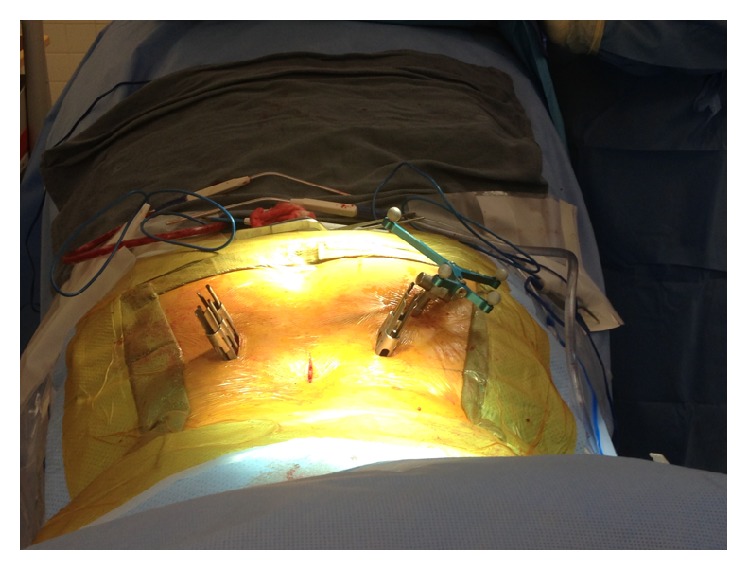
Intraoperative image of a longer segment lumbar fixation case utilizing instrumentation that had low profile reduction MIS towers for ease of rod delivery.

**Figure 9:**
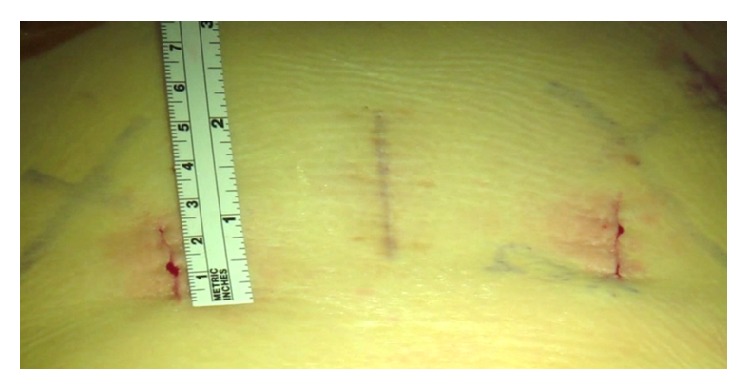
Intraoperative image of the MISS paramedian incision adjacent to a previous midline 2-level laminectomy incision.

In short-segment lumbar fixation, we inserted the rod under direct visualization using a rod holder (Figures 7(a) and 7(b)). For longer segments and degenerative scoliosis cases, we utilized instrumentation that had low profile reduction MIS towers for ease of rod delivery (Figure 8). All patients underwent decortication and dorsal onlay of bone on the lateral lamina, facet, and transverse processes. Final MISS paramedian incisions are demonstrated next to a previous midline 2-level laminectomy incision (Figure 9).

### 2.7. Guide Wires

Management of guide wires during navigated MISS is extremely critical. As there is no ability to navigate the tip of the guide wire in real time, there is a theoretical risk of inadvertently pushing the guide wire through the vertebral body and into the abdominal cavity. In our technique, during navigated MISS, we pay special attention to guide wire location. After a cannulated instrument or screw is started in the proximal pedicle, we recommend pulling the guide wire back several inches. This essentially eliminates the possibility of inadvertent guide wire advancement. “Guide wireless” navigated MISS techniques have also been previously described [24]. The reverse-projection option on the StealthStation computer screen allows for saving of pedicle trajectory without guide wires. However, we found that using guide wires was much more reproducible and reliable and led to faster delivery of pedicle taps, screws, and overall surgery [7–9].

### 2.8. Postoperative Course

Intraoperative confirmatory O-arm images and postoperative CT scans were analyzed for evidence of bony pedicle wall breach. The majority of postoperative CT scans were obtained in the outpatient setting for diagnosis purposes or to confirm evidence of bony fusion. Whenever possible, immediate postoperative CT scans were generally not obtained on asymptomatic patients secondary to concern for undue radiation exposure.

## 3. Discussion

The newest generation of navigation technology—intraoperative computed tomography image-guided navigation (CT-IGN) with the mobile O-arm scanner—has made a tremendous impact on spinal surgery and there is a wealth of literature on the topic [10, 11, 25]. Increasing imaging resolution has led to improved accuracy of instrumentation placement in the thoracic and lumbar spine, primary versus revision cases, which has in turn led to overall superior clinical outcomes [6–9, 12]. Baaj et al. recently published an exclusive study on O-arm technology and its use in MIS spine surgery [25]. They presented 14 cases of complex, multilevel segmental fusions in which a total of 110 screws were placed percutaneously or transfascially. Their mean estimated blood loss (EBL) was minimal at 156 cc and mean operative time was 296 min, which included repositioning time. There were a total of 6 screw breaches (4 lateral and 2 medial) without neurological deficits. They concluded that their technique is practical and consistently reliable at providing insertion accuracy and corresponding improved outcomes, though long-term follow-up is required for confirmation. Cho et al. also studied the O-arm with mini-open TLIF and found 4 pedicle perforations >2 mm in 82 screw insertions in 20 patients without neurological injury [26]. Of special significance, they emphasized the importance of a cutaneous mounted dynamic reference frame in providing accuracy in 3D image-guided navigation, which in their study was over the sacral hiatus.

In a more recent study, Lau et al. investigated computer-guided intraoperative O-arm fluoroscopy in aiding the visualization of pedicle screw placement via minimally invasive transforaminal interbody fusion (MITLIF) [27]. While previous studies reported on the increased accuracy of percutaneous pedicle screw placement in MIS, Lau et al. reported the O-arm's effect on decreasing the incidence of facet violations [27]. Because in minimally invasive techniques screws are inserted percutaneously, there is decreased visualization of the facet joints and thus a risk of facet violation and future adjacent segment disease. While the authors expected lower rates of facet fusion, this was not the case. In fact, although not statistically significant and potentially confounded by higher body mass index (BMI) patients, the O-arm group had greatest facet violations. Adjusting for BMI, the O-arm group was not associated with higher or lower risk of facet violations, although BMI did remain as an independent risk factor [27].

Park et al. were the first to publish a study to evaluate the O-arm alongside minimally invasive pedicle screw placement [3]. In 11 patients with 52 screws, they found a misplacement rate of 7.5% and no breach was greater than 2 mm, numbers that were very similar to previous studies utilizing similar computer-guidance in open spinal procedures. Although a preliminary study in a small number of patients, their technique was found to be comparatively accurate and safe. A year later, Garrido and Wood reported their experience with the O-arm alongside MIS procedures in another region, the lumbopelvic junction [28]. Their goal was a technique for lumbopelvic fixation that would minimize extensive dissection and its associated sequelae, decrease radiation exposure, provide better picture quality, and facilitate the complexity of hardware placement. In the study, 5 patients underwent 10 O-arm guided iliac bolts with neither violations of the sciatic notch nor misplacement of bolts. At the same time, their technique together with the O-arm helped them achieve the goals they set out to accomplish.

Three-dimensional image guidance has also been applied to the cervical spine. Because it offers a vast amount of anatomic information and allows safer approaches around complex anatomy, such technology can be quite helpful for navigating the cervical spine [23]. Though it was not a minimally invasive techniques-based study, Nottmeier and Young published on 3D image guidance for screw placement in the occipitocervical region [29]. Eighty-two screws in 18 patients were placed at C1, C2, or occipital levels without any complications and only a single screw had minimal breach postoperatively. Kim et al. published a series on modified transcorporeal anterior cervical microforaminotomy (MTACM) assisted by O-arm navigation [23]. They presented 8 patients with radicular upper extremity symptoms who underwent this procedure without complications and with postoperative improved symptoms. The combination of a minimally invasive technique and O-arm guidance enabled improving both the accuracy and the outcomes of the procedure. More recently, Del Curto et al. reported on a similar technique of minimally invasive posterior cervical microforaminotomy assisted by O-arm navigation with also similar effective and safe results [30].

Oertel et al. presented 50 patients that underwent spinal stabilization surgeries, 10 of which were through percutaneous pedicle screws [21]. Of the 278 total pedicle screws inserted, only 9 screws breached (all medially, <2 mm) without nerve root injury. They reported that, compared to standard CT-based navigation and Fluoromerge, O-arm-based navigation is the most precise and accurate method. Additionally, their experience indicated that the image quality of the O-arm is almost identical to a CT scan and only in patients with a weight of >250 lbs does the image quality begin to degrade. In terms of ergonomics and workflow, the authors stated that the apparatus is designed to best support a quick setup for easy acquirement of images with minimal technical hurdles and memory robotic movement to quickly relocate to the ideal position [21].

The O-arm's application to MISS procedure was recently described in a major study by Houten et al. [4]. They presented a large clinical series of MIS percutaneous screw placement using O-arm imaging and compared the results with fluoroscopy-guided surgery. All patients in the study underwent minimally invasive lumbar interbody fusion (MIS-TLIF, DLIF, and XLIF) mostly for the treatment of spondylolisthesis. The O-arm group had 52 patients with 205 screws and the fluoroscopy-based group had 42 patients with 141 screws. The perforation rate was 3% versus 12.8% (*P* < 0.001) and the mean operative time was 200 versus 221 minutes (*P* < 0.03), respectively. The study builds upon the results of Oertel et al., reporting similar excellent outcomes with O-arm in MISS cases [21]. Additionally, they found that image quality was maintained even with patients over >300 lbs (maximum 339 lbs). Lastly, they noted that the O-arm allowed the surgeon to place screws without having to wear heavy lead which is required with fluoroscopy.

## 4. Advantages and Disadvantages of the O-Arm System

The cone beam O-arm system offers several advantages over prevailing imaging methods, especially for MISS which heavily benefits from use of navigation. There is the capacity for reduction in radiation exposure to the operating room staff that has been cited extensively in the literature [4, 5, 31, 32]. It was reported that, through an assessment of its dosimetric features, an O-arm scan sends about 50% of the radiation dose of 64-cut CT scan [33]. A second advantage of the cone beam O-arm is its ability to provide a more detailed view of the pedicles that allows for more accurate screw placement. This has been shown in studies to decrease the likelihood of neural injury and negative clinical sequelae [4]. Silbermann et al. also reported that navigation guidance with cone beam CT imaging was significantly more accurate in screw placement when compared with freehand technique, reaching almost 100% [34]. Accuracy of cone beam CT navigation has been packaged with automatic registration and versatility in software to minimize human error and reduce overall operative time. With the final ability to promptly acquire postoperative scans to avoid revision surgery, current technology cone beam CT navigation has evolved to provide the surgeon with numerous invaluable advantages. Disadvantages of current CT image navigation have also been reported. Current capital expenditure costs for the O-arm mobile CT scanner are estimated to be approximately $700,000 and the StealthStation guidance system approximately $250,000. Although costs of new technology are steadily decreasing over time, it is imperative that more research on cost-effectiveness be undertaken in order to financially justify navigation technology [10].
